# CD8^+^ T Cells as a Source of IFN-γ Production in Human Cutaneous Leishmaniasis

**DOI:** 10.1371/journal.pntd.0000845

**Published:** 2010-10-12

**Authors:** Mahmoud Nateghi Rostami, Hossein Keshavarz, Rosita Edalat, Abdolfattah Sarrafnejad, Tahereh Shahrestani, Fereidoun Mahboudi, Ali Khamesipour

**Affiliations:** 1 Medical Parasitology and Mycology Department, School of Public Health, Tehran University of Medical Sciences, Tehran, Iran; 2 Faculty of Health, Qom University of Medical Sciences, Qom, Iran; 3 Biotechnology Department, Pasteur Institute of Iran, Tehran, Iran; 4 Immunology Department, School of Public Health, Tehran University of Medical Sciences, Tehran, Iran; 5 Center for Research and Training in Skin Diseases and Leprosy, Tehran University of Medical Sciences, Tehran, Iran; Institut Pasteur, France

## Abstract

**Background:**

In human leishmaniasis Th1/Th2 dichotomy similar to murine model is not clearly defined and surrogate marker(s) of protection is not yet known. In this study, Th1/Th2 cytokines (IL-5, IL-10, IL-13 and IFN-γ) profile induced by purified CD4^+^/CD8^+^ T cells in response to *Leishmania* antigens were assessed at transcript and protein levels in 14 volunteers with a history of self-healing cutaneous leishmaniasis (HCL) and compared with 18 healthy control volunteers.

**Methodology/Principal Findings:**

CD4^+^/CD8^+^/CD14^+^ cells were purified from peripheral blood using magnetic beads; CD4^+^/CD8^+^ T cells were co-cultured with autologous CD14^+^ monocytes in the presence of soluble *Leishmania* antigens (SLA). Stimulation of either CD4^+^ T cells or CD8^+^ T cells of HCL volunteers with SLA induced a significantly (*P*<0.05) higher IFN-γ production compared with the cells of controls. Upregulation of IFN-γ gene expression in CD4^+^ cells (*P*<0.001) and CD8^+^ cells (*P* = 0.006) of HCL volunteers was significantly more than that of controls. Significantly (*P*<0.05) higher fold-expression of IFN-γ gene was seen in CD4^+^ cells than in CD8^+^ cells. In HCL volunteers a significantly (*P* = 0.014) higher number of CD4^+^ cells were positive for intracellular IFN-γ production than CD8^+^ cells.

**Conclusions/Significance:**

Collectively, the volunteers have shown maintenance of specific long-term immune responses characterized by a strong reaction to leishmanin skin test and IFN-γ production. The dominant IFN-γ response was the result of expansion of both CD4^+^ and CD8^+^ T cells. The results suggested that immune response in protected individuals with a history of zoonotic cutaneous leishmaniasis (ZCL) due to *L. major* is mediated not only through the expansion of antigen-specific IFN-γ producing CD4^+^ Th1 cells, but also through IFN-γ producing CD8^+^ T cells.

## Author Summary

Cutaneous leishmaniasis (CL) is usually a self-healing skin lesion caused by different species of *Leishmania* parasite. Resistance and susceptibility of mice to *Leishmania major* infection is associated with two types of CD4+ T lymphocytes development: Th1 type response with production of cytokine IFN-γ is associated with resistance, whereas Th2 type response with production of cytokines IL-4 and IL-5 is associated with susceptibility. A clear Th1/Th2 dichotomy similar to murine model is not defined in human leishmaniasis and we need as much information as possible to define marker(s) of protection. We purified CD4^+^/CD8^+^ T cells, stimulated them with *Leishmania* antigens and analysed gene and protein expression of Th1/Th2 cytokines in volunteers with a history of self-healing CL who are presumed to be protected against further *Leishmania* infection. We have seen significant upregulation of IFN-γ gene expression and high IFN-γ production in the *Leishmania* stimulated CD4^+^ T cells and CD8^+^ T cells. We concluded that both antigen-specific IFN-γ producing CD4^+^ Th1 cells and IFN-γ producing CD8^+^ T cells contribute to the long term protection in individuals with a history of CL. This proves the importance of CD8+ T cells as a source of IFN-γ in Th1-like immune responses.

## Introduction

Leishmaniasis is expanding both by increasing the incidence rate in endemic foci and extending the disease to new regions [1], [2]. Control measures against leishmaniasis are not fully effective, chemotherapy is not always successful, and drug resistant is emerging [3]–[5]. Although theoretically development of an effective vaccine against leishmaniasis is feasible but yet there is no vaccine available against any form of leishmaniasis [6], [7]. CD4^+^ T cells upon activation differentiate into functional effector Th1 and/or Th2 subsets and the outcome of *Leishmania major* infection in murine model is dependent upon the type of immune response generated: in most strains of mice *L. major* infection induces a Th1 type of response associated with a high level of IFN-γ, low level of IL-4, and similar to human cutaneous leishmaniasis the lesion(s) heals spontaneously and the animals are protected against further infection; whereas *L. major* infection in BALB/c mice induces a Th2 response and a high level of IL-4 and low level of IFN-γ, as a result the disease is fetal [8], [9]. The mechanism(s) of protection in human leishmaniasis is not well characterized; however, the role of T lymphocytes and Th1/Th2 cytokine profile are extensively studied [10]–[16]. In human leishmaniasis, peripheral blood mononuclear cells (PBMC) are routinely collected from patients with different clinical pictures of cutaneous leishmaniasis (CL) for immunological investigations. Results from the majority of these studies showed that PBMC of healing or cured cases of CL produce significant amount of IFN-γ in response to *Leishmania* antigens [17], [18]. There is evidence demonstrating CD4^+^ T cells collected from patients with CL or mucocutaneous leishmaniasis (ML) or individuals with history of CL produced a high level of IFN-γ in response to *Leishmania* antigens which is an indication of a Th1 like response [10], [11]; Conversely, T cells from patients with diffuse CL (DCL) failed to express IL-2 receptor and did not produce IFN-γ in response to *Leishmania* antigens, whereas IL-4 mRNA markedly increased in DCL lesions [17], [18]. A clear Th1/Th2 dichotomy similar to murine model is not yet defined in human leishmaniasis [19].

There are reports which showed that CD8^+^ T cells play a role in controlling intracellular pathogens including protozoal and viral infections. CD8^+^ T cells are shown to confer a significant role in protection against acute and chronic form of *Toxoplasma gondii* infection [20]. In early stage of murine toxoplasmosis, CD8^+^ T cells hamper parasite dissemination by either direct lysis of infected cells or through release of cytokines. During chronic infection CD8^+^ T cells limit *Toxoplasma* cyst formation in tissues [21], [22]. Immunity against malarial sporozoites is mediated partially by neutralizing antibodies, but largely depends on antigen specific CD8^+^ T cells, thus vaccines are designed based on induction of infection-blocking CD8^+^ T cells [23], [24]. CD8^+^ T cells are also important in the control of HIV infection [25]–[27]. During HIV infection, CD8^+^ T cells recognize infected cells through an MHC-I dependent process and viral infected cells are lysed by secretion of perforin and granzymes [28]. Most patients chronically infected with HIV show CD8^+^ T cell response against HIV virus, but the response is not enough to successfully control viral replication [25]–[27]. In *Listeria monocytogenes* infection, both CD4^+^ and CD8^+^ T cells contribute in induction of protection, but the major bactericidal role is attributed to CD8^+^ T cells [29]. In experimental models of leishmaniasis, CD8^+^ T cells, in cooperation with CD4^+^ T cells, appear to be involved in the induction of host immunity against both primary infection and reinfection of *Leishmania* parasite [30]–[32]. In *L. major* infected CD8^+^ depleted BALB/c mice, during lesion healing the frequency of IFN-γ producing CD4^+^ T cells and the amount of IFN-γ are diminished resulted in a higher parasite burden [30]. In a study performed on C57BL/6 mice, infection with low dose of *L. major* induces a transient Th2 type response and then shifts to a Th1 response associated with healing. Induction of this Th1 type of response partly depends on the activation of IFN-γ producing CD8^+^ T cells and in the absence of CD8^+^ T cells, the Th2 response is sustained [33]. In mouse model of both genetically resistant and susceptible (that were rendered resistant) backgrounds, CD8^+^ T cells have been demonstrated to produce IFN-γ and contribute to the rapid healing of secondary lesions which develop after primary challenge with *L. major* [31].

There are reports from New World leishmaniasis which showed that CD8^+^ T cells are involved in healing process of CL due to *L. braziliensis* [34]–[37]. However, to our knowledge there is no data available about the possible role of CD8^+^ T cells and their cytokines in CL due to *L. major*.

In leishmaniasis, most of the data generated so far is drawn from PBMCs culture without separation of T cell subtypes [10], [12], [13]which makes it difficult to judge the role of Th1/Th2 CD4^+^ cells and CD8^+^ T cells. In the current study two major lymphocyte subtypes, CD4^+^ and CD8^+^ T cells, were purified from individuals with history of self-healing CL and cytokine pattern were analyzed at transcript and protein levels in response to *Leishmania* antigens.

## Materials and Methods

### Study population and ethical considerations

The study was approved by Ethical Committee of Tehran University of Medical Sciences. Potential candidates were invited and those who were willing to participate and sign a written informed consent were recruited.

Fourteen volunteers with history of self-healing CL (HCL) caused by *L. major* and with leishmanin skin test (LST) more than zero and as control 18 healthy volunteers from non-endemic area with no response to LST were included. HCL volunteers were selected among the previous Center's patient who received no treatment for the CL lesion(s) and the lesion(s) cured spontaneously within one year of onset. The causative agent of every CL patient was previously identified as *L. major* using PCR method.

### Soluble *Leishmania* antigen preparation

*Leishmania major* (MRHO/IR/75/ER) was cultured on NNN medium and passaged on RPMI 1640 (Gibco Invitrogen, Carlsbad, CA, USA) supplemented with 10% fetal calf serum (FCS). Promastigotes were harvested at day 5, washed 3 times with PBS (pH 7.2) and used for preparation of soluble *Leishmania* antigen (SLA) as previously described [14]. Briefly, protease inhibitor cocktail enzyme (Sigma, St. Louis, MO, USA) was added at 100 µl per 1×10^9^ promastigotes, and then the parasites were freeze-thawed 10 times followed by sonication at 4°C with two 20-sec blasts. Parasite suspension was centrifuged at 30,000×g for 20 min, the supernatant was collected and re-centrifuged at 100,000×g for 4 hours. SLA protein concentration was measured using Bradford method [38]. Finally the supernatant was sterilized using 0.22 µm membrane filter, aliquoted and stored at −20°C until use.

### Purification of CD4^+^/CD8^+^/CD14^+^ cells

Twenty mL of blood sample was collected from each volunteer and Peripheral Blood Mononuclear Cells (PBMCs) were isolated using Ficoll–Hypaque (Sigma, St. Louis, MO, USA) density gradient centrifugation. CD4^+^ and CD8^+^ lymphocytes isolation was performed using magnetic beads system (StemCell Technologies Inc., Vancouver, BC, Canada) by positive selection using anti-CD4 or anti-CD8 coated nanoparticles. Briefly, cell suspension was prepared at a concentration of 1×10^7^ cells/ml in a 5 ml tube in isolation buffer containing PBS plus 2% (v/v) FBS and 1 mM EDTA. EasySep CD4/CD8 cocktail Abs was added at 10 µl/ml cells, mixed well and incubated at room temperature (RT) for 15 min. Magnetic nanoparticles were added at 5 µl/ml cells and incubated for 10 min at RT. The cell suspension was brought to 2.5 ml by adding buffer and the tube was placed into the magnet for 5 min, then the supernatant was discarded. The desired cells were remained bound inside the tube. The steps of placing tube into the magnet were repeated three times.

Monocytes (CD14^+^) were isolated from autologous PBMC by negative selection according to the manufacturer's instruction (StemCell Technologies Inc., Vancouver, BC, Canada). Briefly, cell suspension was prepared at a concentration of 5×10^6^ cells/ml in isolation buffer. EasySep monocyte enrichment cocktail Abs was added at 5 µl/ml cells, mixed well and incubated at 4°C for 10 min. Magnetic microparticles were added at 5 µl/ml cells for 5 min at 4°C. The cell suspension was brought to 2.5 ml by adding buffer and the tube was placed into the magnet, for 2.5 min at RT. The desired unbound fraction was transferred into a new tube.

**Figure 1:**
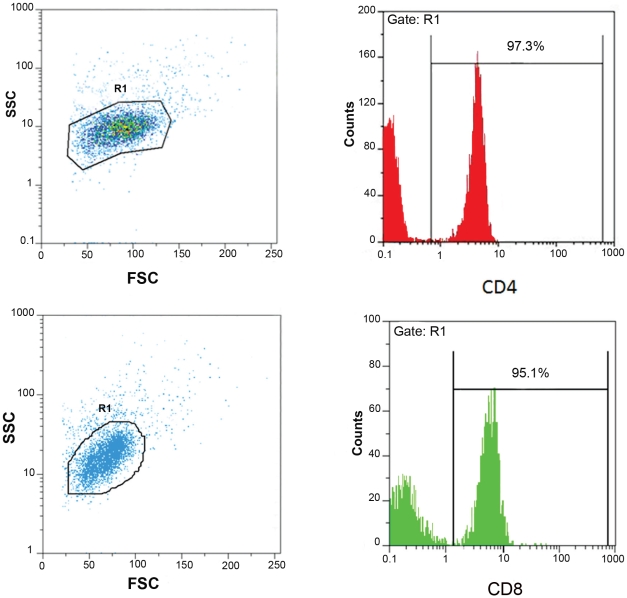
The purity of peripheral blood enriched CD4^+^ and CD8^+^ T cell populations after magnetic beads isolation. CD4^+^ (A) and CD8^+^ (B) lymphocytes were isolated from a cell suspension of PBMC by positive selection using CD4/CD8 cocktail Abs and anti-CD4 or anti-CD8 coated magnetic nanoparticles. The purity of yielded T cell populations was analysed by flow cytometry using conjugated mAbs.

The purity of the yielded lymphocytes or monocytes was more than 95% by flow cytometry analysis using specific conjugated mAb (Fig. 1). The contamination of CD8^+^ T cells with NK cells was less than 9% using α-CD56 mAb.

### Purified CD4^+^/CD8^+^ T cell culture

Monocytes were co-cultured with sorted lymphocytes as antigen presenting cells (APCs) following mitomycin C (Merk, Darmstadt, Germany) treatment at a final concentration of 10 µg/ml for 30 min at 37°C with 5% CO2.

The cells were cultured in RPMI 1640 media supplemented with 10% heat-inactivated human AB Rh+ serum, 10 mM/L Hepes, 2 mM L-glutamine, 100 U/ml penicillin G and 100 µg/ml streptomycin (Gibco Invitrogen, Carlsbad, CA, USA). CD4^+^ or CD8^+^ lymphocytes were adjusted to 0.5–1×10^6^ cells/ml mixed with 1∶10 of autologous monocytes and were cultured in U-bottomed 96-well plates (Nunc, Roskilde, Denmark) in the presence of either 10 µg/ml PHA or 50 µg/ml of SLA in a final volume of 200 µl. Plates were incubated at 37°C with 5% CO2 in humidified atmosphere for 72 hrs.

### ELISA cytokine assay

Culture supernatants were collected at 72 hours, the level of IL-5, IL-10, IL-13 and IFN-γ were titrated in culture supernatants using ELISA method (Mabtech, Stockholm, Sweden). Briefly, the plates were coated with anti-IFN-γ/IL-5/IL-10/IL-13 mAb in PBS, pH 7.4, and incubated at 4°C over night. After blocking the wells using buffer containing PBS plus 0.05% (v/v) Tween 20 and 0.1% (w/v) BSA, supernatants were added to each well. Biotin-labeled mAb in incubation buffer was added to each well and as enzyme streptavidin-HRP was used. The reaction was developed using 3,3′,5,5′-tetramethylbenzidine (TMB) substrate and stopped with 0.5M H2SO4 solution. The plates were washed after each step of incubation using PBS+0.05% (v/v) Tween20. The plates were read at 450 nm using a reader (BioTek, Winooski, VT, USA). The mean optical densities (ODs) of triplicate cultures were compared with the standard curves prepared using recombinant IL-5, IL-10, IL-13 and IFN-γ. The cytokine levels represent the differences between the ODs of test and background wells. The detection limit of the assays was 4 pg/ml for IL-5 and 0.5 pg/ml for IL-10, 5 pg/ml for IL-13 and 2 pg/ml for IFN-γ.

### Intracellular Cytokine Staining (ICS)

After SLA stimulation, part of the cells was used for ICS assay. Cells were adjusted at 5×10^5^ per ml and stimulated with PMA (Sigma, St. Louis, MO, USA) 50 ng/ml plus Ionomycin calcium (Sigma) 500 ng/ml and incubated at 37°C, 5% CO2 for 5–6 hrs. Monensin (Sigma) was added at 25 µM/ml during the last 4–5 hrs of culture for blocking. Cells were harvested and washed 2 times with PBS (pH 7.2) plus 0.1% bovine serum albumin (BSA). The cells were permeabilized using BD Cytofix/Cytoperm kit according to the manufacturer's instruction (BD Biosciences, San Jose, CA, USA). In the final step, cells were stained with FITC-conjugated mouse anti-human IFN-γ and PE-conjugated rat anti-human IL-2 (BD Biosciences, San Jose, CA, USA). Cells were washed ×2 with perm/wash buffer and resuspended in PBS (pH 7.2) plus 1% BSA. Cells were analyzed using Partec flow cytometer (DAKO cytomation, Glostrup, Denmark) while isotype matched negative controls were used to set the threshold of autofluorescence. A minimum of 50,000 events were acquired for each sample. FACS data analysis was performed using FloMax (DAKO cytomation) software.

### RNA extraction

At the time of supernatants collection (at day 3), the SLA stimulated cells were harvested and used for RNA extraction. The procedure began with reverse transcription of mRNA to cDNA. The cDNA was then used as template for Real-time PCR using specific primer of each cytokine. Solutions were treated and glassware was filled with 0.1% (v/v) diethylpyrocarbonate (DEPC) (Merck, Darmstadt, Germany) in H2O.

The cell pellet was resuspended in cold PBS (pH 7.2) and lysed by addition of 0.2 mL of Trizol (Sigma, St. Louis, MO, USA) per 1×10^6^ cells. RNA was solublized through pipetting and incubated at RT for 5 min. Then 0.2 ml chloroform was added per 1 ml of homogenate, the suspension was shook vigorously and kept on ice for 5 min followed by centrifugation for 15 min, 12,000×g at 4°C. The upper phase was collected and added to an equal volume of isopropanol and incubated at 4°C over night. Then the cell suspension was centrifuged at 12,000×g, 4°C for 15 min, the supernatant was discarded and the RNA pellet was washed with 1 ml 75% ethanol at 7,500×g for 8 min. At the end, the pellet was drayed briefly and dissolved in DEPC treated water. The purity of RNA samples was assessed by the ratio of ODs at 260/280 nm using UV spectrophotometry.

### Real-time PCR

Reverse transcription was carried out using RevertAid M-MuLV enzyme (Fermentas life sciences, York, UK) in a 30 µL reaction mixture. Briefly, 1 µL (0.5 µg) of oligo dT_18_ primer was added to about 3 µg of total RNA, mixed and incubated at 70°C for 5–10 min. The tube was placed on ice for a few minutes, centrifuged briefly and added with: 4 µL of 5× reaction buffer, 1 µL 10 mM dNTPs, 20 u RNase inhibitor (RiboLock; Fermentas life sciences) and DEPC treated water up to 30 µL. Tube was incubated at 37°C for 5 min, the contents were mixed gently and then added with 100 U of enzyme. The reaction mixture was incubated at 42°C for 60 min. The enzyme was inactivated by heating at 70°C for 10 min. and chilled on ice. In a Real-time PCR MicroAmp optical 96-well reaction plate (Applied Biosystems, Foster City, CA, USA) for 25 µl reaction mixture the followings in each optical well were prepared: 12.5 µl QuantiTect SYBR Green I (Qiagen, Hilden, Germany), 3 µl cDNA, 2 µl primer pair mix (0.5 µM each), 7.5 µl dH2O (For sequences of primer pairs see Table 1).

**Table 1:** Sequence of primer pairs for different amplicons.

GENE	FORWARD	REVERSE	PRODUCT SIZE (bp)
**IFN-γ**	IFN-TGTCCAACGCAAAGCAATAC	TCGACCTCGAAACAGCATCT	106
**IL-5**	CTTGGCACTGCTTTCTACTCATC	TGCAGGTAGTCTAGGAATTGGTT	261
**IL-10**	GCCGTGGAGCAGGTGAAG	AGTCGCCACCCTGATGTCT	144
**IL-13**	AACATCACCCAGAACCAGAAG	CAGAATCCGCTCAGCATCC	158
**GAPDH**	GAAGGTGAAGGTCGGAGTC	GAAGATGGTGATGGGATTTC	226

For normalizing the difference in the amount of inputting cDNAs, the housekeeping gene glyceraldehyde-3-phosphate dehydrogenase (GAPDH) was used as the internal standard. Samples and internal standard were amplified in separate wells of the plate. Two-step thermal profile as a PCR program was set up on the SDS software (version 1.3.1) of Applied Biosystems 7,500 machine (Applied Biosystems, Foster City, CA, USA).
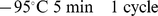


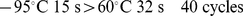
The dissociation curve on the instrument software was set as follows:
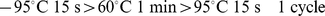
Software generates reports including amplification plots and dissociation curves. Any bimodal dissociation curve or abnormal amplification plots were checked to see if there is an indication of different Tms and nonspecific products.

The Ct (threshold cycle) of each sample was used in gene relative expression calculation. The 2^−ΔΔCt^ method was used to calculate relative changes in the gene expressions:
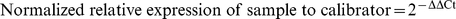
While
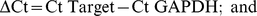


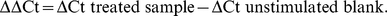



### Efficiency assay: Validation of ΔΔCT method

To have a valid calculation of 2^−ΔΔCt^, the amplification efficiencies of the target and reference (GAPDH) genes must be approximately equal. For this purpose, the Ct values variation with cDNA template dilutions was checked. A pooled cDNA preparation was diluted over a 10-fold range and PCR was performed for each dilution using specific primers. A plot of the log cDNA dilution versus CT was prepared. The slope of each line was obtained from regression equation and the efficiencies of the target and reference genes were calculated using the equation: Efficiency (E) = [10 ^(1/slop)^]−1

### Statistical analysis

Non-parametric tests of Mann-Whitney, Kruskal-Wallis and Dunn's post-test for paired comparisons were used for statistical analysis of the data using SPSS version 11.5 (SPSS Inc., Chicago, IL, USA) and GraphPad Prism version 5.01 (GraphPad Software Inc., La Jolla, CA, USA) softwares. Nonparametric tests were chosen because the samples did not follow a Gaussian distribution. P value of <0.05 considered to be significant.

## Results

Basic information of the studied population is outlined in Table 2.

**Table 2:** Basic information of the volunteers.

Characteristic	HCL	Control
**Sex: M/F**	9/5	12/6
**Age**	29±10.5	34.9±6.7
**Number of lesion**	1 (1–10)	NA
**Duration of the lesion(s) (Months)**	3 (1.5–5)	NA
**Time between healing and sampling (Months)**	1 (1–120)	NA
**Leishmanin Skin Test (mm)**	10.7±7.48	0

Age and LST: Mean±S.D.

Others: Median (Range).

NA = not applicable.

### ELISA for cytokine production assay for CD4^+^/CD8^+^ T cells

**Figure 2:**
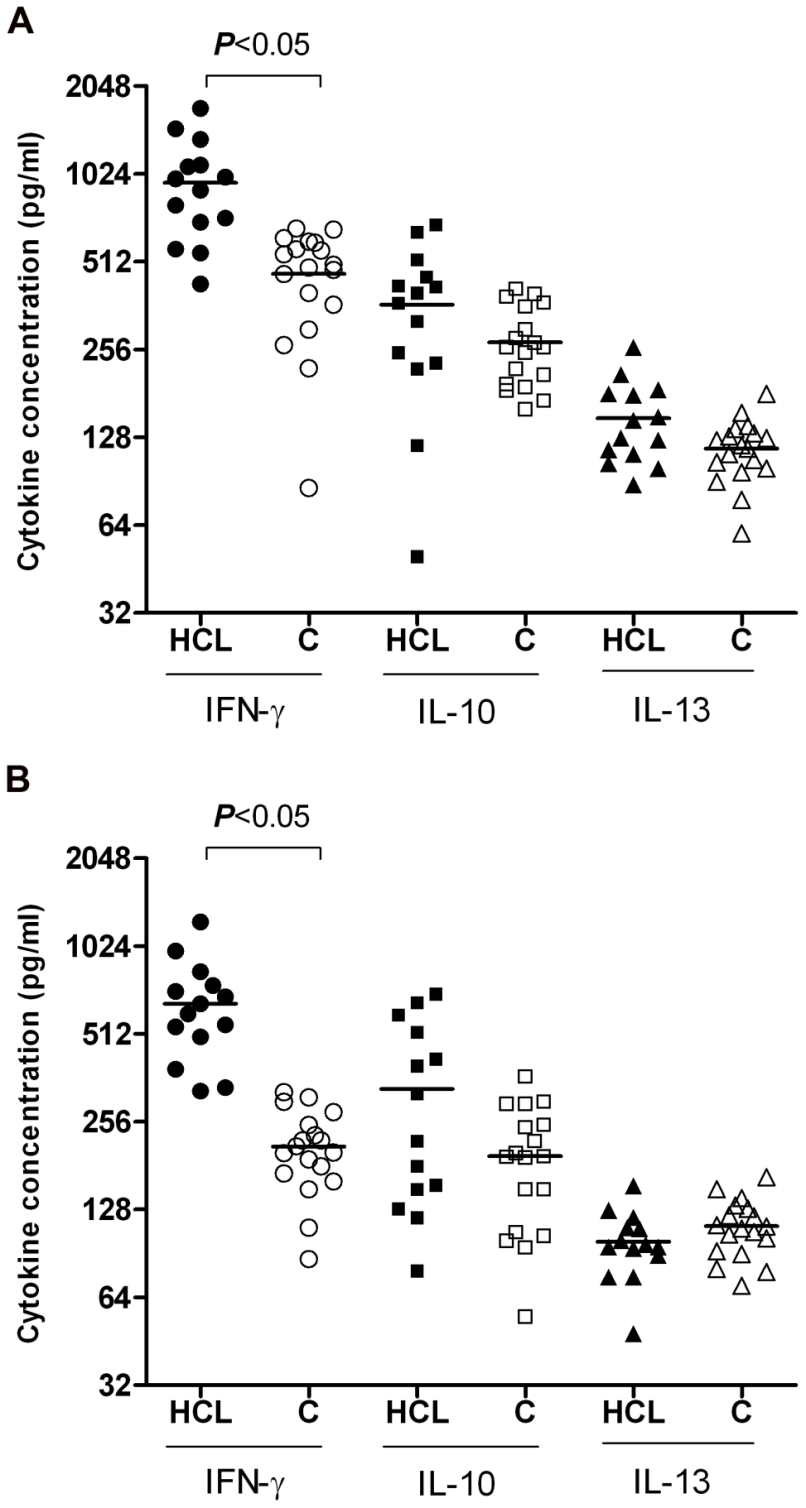
Cytokine profile of CD4^+^ and CD8^+^ T cells after SLA stimulation. Purified CD4^+^ and CD8^+^ T cells were adjusted to 1–2×10^5^ cells/well in a U-bottomed 96-well plates and co-cultured with 1∶10 of mitomycin treated autologous monocytes in cRPMI 1640 supplemented with 10% human AB Rh+ serum. IFN-γ, IL-5, IL-10, and IL-13 were titrated on supernatant of SLA stimulated of CD4^+^ (A) and CD8^+^ (B) T cells at 72 hrs of culture using sandwich ELISA method. The amount of IL-5 was not detectable in the culture supernatants. Filled symbols represent HCL volunteers; Open symbols represent healthy controls.

Using ELISA method, cytokine profile (IFN-γ, IL-10, IL-13) were measured on supernatants collected at 72 hrs of SLA stimulated PBMC or CD4^+^, CD8^+^ T cells culture. The amount of IFN-γ level was significantly higher in PBMC culture of HCL volunteers compared with that of healthy controls (*P*<0.05). Results of purified T cell culture showed that stimulated CD4^+^ T cells from HCL volunteers induced a significantly higher IFN-γ production compared with cells from healthy controls (*P*<0.05) (Fig. 2A). Similarly, stimulated CD8^+^ T cells from HCL volunteers induced a significantly higher IFN-γ production compared with the cells from healthy controls (*P*<0.05) (Fig. 2B). The levels of IL-10 and IL-13 were not significantly different in either CD4^+^ or CD8^+^ T cells between HCL volunteers and healthy controls (Fig. 2A and B). IL-5 level was not detectable in culture supernatants of either CD4^+^ or CD8^+^ T cells.

### Real-time PCR for cytokine mRNA expression assay in CD4^+^/CD8^+^ T cells

**Figure 3:**
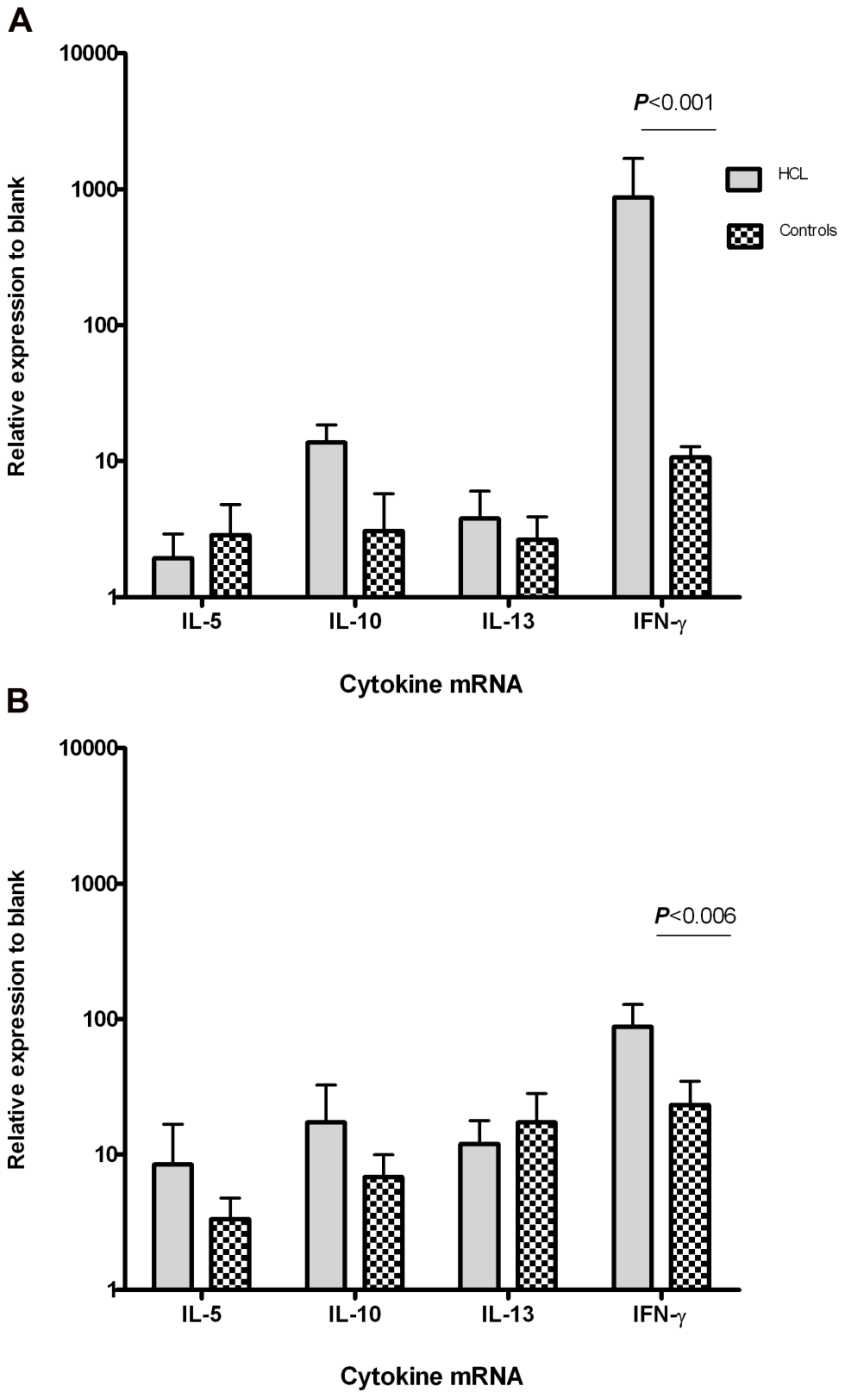
Relative expression of cytokine genes in SLA stimulated CD4^+^ and CD8^+^ T cells to unstimulated cells (blank wells) of culture. Total RNA was extracted from stimulated CD4^+^ and CD8^+^ T cells of culture and reverse transcription of mRNA to cDNA was performed using M-MuLV enzyme. Two steps Real-time PCR was set on cDNA samples using SYBR Green I system and specific primer pairs. Threshold cycles (Cts) of each amplicon was used for further analysis. The relative quantities of the target genes were normalized against the relative quantities of the internal standard (GAPDH). Fold-expression changes were calculated using the equation 2^−ΔΔCT^. A) relative expression of cytokine genes in CD4^+^ T cells B) relative expression of cytokine genes in CD8^+^ T cells.

The relative quantities of the target genes were normalized against the relative quantities of the internal standard (GAPDH). Ct values of amplified templates of antigenic stimulated T cells were used for calculation of different cytokine gene expressions using 2^−ΔΔCT^ method. The expression amount was compared with unstimulated cells of culture and relative fold-expression was reported. Result for each donor was calculated and then data was pooled and presented as a mean of HCL volunteers against healthy controls. Results showed that the upregulation of IFN-γ gene expression in CD4^+^ cells from HCL volunteers was significantly higher than that of healthy controls (*P*<0.001) (Fig. 3A). Similarly, fold-expression changes of IFN-γ gene was significantly higher in CD8^+^ cells from HCL volunteers compared to the cells from healthy controls (*P* = 0.006) (Fig. 3B). Comparing CD4^+^ and CD8^+^ T cells, the significantly higher fold-expression of IFN-γ gene was seen in CD4^+^ cells than CD8^+^ cells of HCL volunteers.

In both CD4^+^ and CD8^+^ T cell cultures, the changes in the gene expression of IL-5, IL-10 and IL-13 were not significantly different between HCL volunteers and healthy controls.

By amplification of serially diluted pooled cDNA, the amplification efficiency of the target (cytokines) compared to reference (GAPDH) genes was examined using SYBR Green detection. Using the equation pointed out in methods, efficiency of GAPDH was 96% while that of targets were between 91% and 95%.

### Intracellular cytokine staining (ICS)

**Figure 4:**
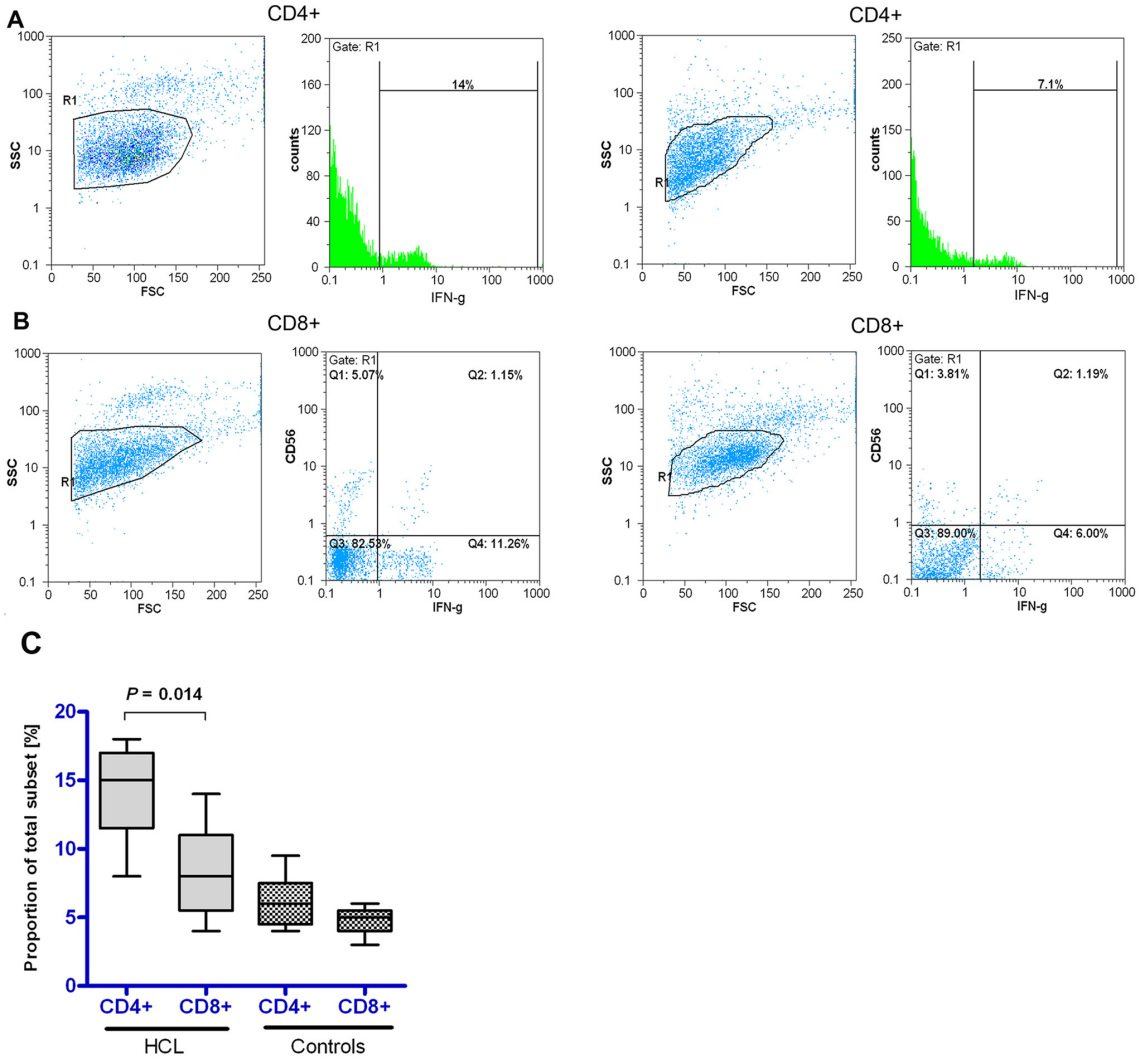
Frequency of purified CD4^+^/CD8^+^ T cells producing intracellular IFN-γ. A portion of the cells at 72 hrs of SLA stimulation was used for ICS assay. Cells were adjusted at about 5×10^5^/ml and stimulated with PMA + Ionomycin for 5–6 hrs. Monensin was added during the last 4–5 hrs of culture. Cells were permeabilized and stained for intracellular IFN-γ with conjugated mAbs. A) One representative flow cytometry plot showing intracellular IFN-γ positive fractions of gated populations of CD4^+^ and CD8^+^ T cells in HCL volunteers. B) One representative flow cytometry plot showing intracellular IFN-γ positive fractions of gated populations of CD4^+^ and CD8^+^ T cells in healthy controls. C) Flow cytometry data of all volunteers were pooled and are shown as Median (horizontal line) with interquartile ranges (box) and range (whiskers) of intracellular IFN-γ positive cells.

Seventy two hrs after SLA stimulation of CD4^+^/CD8^+^ T cells, part of the cells were harvested and stimulated with PMA plus Ionomycin for 5–6 hrs, stained for intracellular IFN-γ with conjugated mAbs and the frequency of positive cells was analyzed using flow cytometry. In CD8^+^ cells compartment, antibody to CD56 marker allowed to exclude IFN-γ positive populations of natural killer cell sources (Fig. 4 A and B). Results of analysis of cells from HCL volunteers and healthy controls were pooled separately and presented as median number of intracellular IFN-γ positive CD4^+^ and CD8^+^ T cells (Fig. 4C). Based on this analysis, HCL volunteers showed that a significantly higher number of CD4^+^ T cells were positive for intracellular IFN-γ production than CD8^+^ cells (*P* = 0.014).

## Discussion

Resistance and susceptibility to *L. major* infection in murine model depend upon induction of Th1 or Th2 response, respectively [8], [9], [39]. Recovery from CL usually is accompanied with long lasting protection and strong immune response generation indicated by *in vivo* LST and *in vitro* lymphocyte response to *Leishmania* antigens [10], [12], [13], [19], [40], yet in human leishmaniasis the surrogate marker(s) of protection is not well defined. Most of the studies performed on human immune response against leishmaniasis is carried out on crude PBMCs without purifying CD4^+^ T cell and CD8^+^ population and there is no report to show a clear-cut CD4^+^ Th1/Th2 response [34]. In the current study cytokines patterns of CD4^+^ Th1/Th2 and CD8^+^ T cells in volunteers recovered from CL is evaluated at the transcript and protein levels.

The results of soluble *Leishmania* antigens (SLA) stimulated cells showed that pure CD4^+^ T cells induced a significantly higher IFN-γ production in HCL volunteers compared to that of healthy controls, IL-5 as a Th2 type cytokine was not detectable and IL-10 and IL-13 levels were not significantly different in culture of T cells from HCL volunteers compared with that of healthy controls. At the same time, the level of cytokines' mRNA expression was evaluated to detect T cell cytokine responses to *Leishmania* stimulation at transcript level, after several experiments to explore the optimum stimulation time points for mRNA analysis. Simultaneous analysis of cytokines gene expression showed a strong up-regulation of Th1 cytokine IFN-γ mRNA in CD4^+^ T cells. In line with the results of secreted proteins, level of Th2 cytokines transcripts including IL-5, IL-10 and IL-13 showed no significant increase in HCL volunteers compared with healthy control. The up-regulation of IFN-γ transcripts expression in *Leishmania* stimulated CD4^+^ T cells consistent with IFN-γ secretion is an indication of Th1 type of response in HCL volunteers parallel with no Th2 response indicating by low level of IL-5, IL-10 and IL-13 cytokines.

Analysis of cytokine secretion and transcript expression of SLA stimulated CD8^+^ T cells showed also a significantly higher IFN-γ production in HCL volunteers compared to the healthy control volunteers. Similar to CD4^+^ T cells, the levels of IL-10 and IL-13 were not significantly different in CD8^+^ T cell culture between HCL volunteers and healthy controls. When CD4^+^ and CD8^+^ T cell response was compared, the level of IFN-γ secretion in SLA stimulated cells was not significantly different between CD4^+^ and CD8^+^ cells, but real-time PCR analysis revealed that expression level of IFN-γ mRNA was higher in CD4^+^ T cells than CD8^+^ T cells. To confirm the real-time PCR results, part of CD4^+^/CD8^+^ sorted T cells were harvested following SLA stimulation and intracellular production of IFN-γ was assessed using flow cytometry. Results of intracellular cytokine staining (ICS) in HCL volunteers confirmed that a significantly higher number of CD4^+^ T cells produced intracellular IFN-γ compared with CD8^+^ T cells (median = 15% *vs.* 8%). Based on the results it seems that the source of IFN-γ production is both CD4+ Th1 cells and CD8+ cells in individuals with history of CL.

The role of CD8^+^ T cells in human *Leishmania* infection is not well known and existed reports are controversial. In a study performed on Sudanese individuals it was suggested that IFN-γ production is associated with CD4^+^ T cells rather than CD8^+^ T cells in individuals with history of CL due to *L. major* [40]. A report from New World leishmaniasis showed that in both asymptomatic and antimonial treated CL individuals caused by *L. braziliensis*, a higher proportions of CD4^+^ than CD8^+^ T cells was present [34]. In another report the authors showed that after treatment of CL due to *L. braziliensis*, the frequency of CD4^+^ and CD8^+^ T cells was the same with approximately constant production of IFN-γ [41]. On the other hand, some clinical studies reported high numbers of *Leishmania* specific CD8^+^ T cells in the lesions and peripheral blood during acute phase and healing process in *L. major* or *L. braziliensis* CL patients [42], [43]. In mice, the requirement of CD8^+^ T cells for the control of *L. major* infection is shown to be partly dependent on the procedure of challenge: β2-microglobuine or CD8^+^ deficient C57BL/6 mice when challenged with high dose of *L. major* have the ability to cure the lesion, which indicates that CD8^+^ T cells are not necessary for the control of primary [44] infection, while in the intradermal challenge with low dose (100 metacyclic promastigotes into the ear dermis) the outcome of primary *L. major* infection in anti-CD8 Ab treated or CD8 deficient mice was dependent on the CD8^+^ T cells [45]. The role of CD8^+^ T cells was studied in CBA and anti-CD4 mAb treated BALB/c mice healed from *L. major* infection. The cured mice were re-challenged with *L. major* in the contralateral footpad and lymph nodes cells were depleted of CD4^+^ T cells and stimulated *in vitro*. The remaining CD8^+^ T cells produced a significant amount of IFN-γ [31]. It is believed that in the resolution of the primary *Leishmania* infection and induction of protection in murine model CD8^+^ T cells play an important role [31].

In the present study, following the isolation of CD4+/CD8+ T cells, Th1/Th2 cytokines were titrated on culture supernatant of *in vitro* restimulated T cells to check the type of immune response elicited against *Leishmania* antigen. The main cytokine produced was found to be IFN-γ in the volunteers' T cells. IFN-γ eliminates intracellular pathogens primarily through macrophage activation. Macrophages upon activation produce nitric oxide (NO) which inhibits growth of intracellular pathogens. It is shown that during active lesion of CL due to *L. major* the proliferative response and IFN-γ production of PBMC was increased [12], [46] and T cells from healed CL produced a significantly higher level of IFN-γ but a low level of IL-10 than the cells from controls [14], [15], [40]. Similarly, studies in *L. braziliensis* infection demonstrated a Th1/Th2 mixed response in early stage of active CL [11], [42] and then a sustained Th1 response with elevated level of IFN-γ and down-regulation of IL-4 and IL-10 production were seen apparently associated with healing [11]. Likewise, the presence of high level of IFN-γ in the skin lesions of CL patients support the role of IFN-γ in healing process [46]. Using RT-PCR, the cytokine patterns of skin lesions of CL patients showed predominance IFN-γ, and low levels of IL-5 and IL-10 [17]. The contribution of IFN-γ to the recall of immunological memory against *L. major* reinfection was assessed in mice. The neutralization of IFN-γ at the time of reinfection reduced the specific DTH response, showing the involvement of IFN-γ in the recall of memory response to *L. major* [31]. Similarly in intracellular infection with *T. gondii*, it is shown that CD8^+^ T cells confers resistance against acute infection [20] and IFN-γ producing CD8^+^ T cells play a significant role in controlling chronic *T. gondii* infection and inhibits encephalitis in mouse model [21], [22].

In the current study, even though using real-time PCR the expression level of IFN-γ transcripts in CD8^+^ cells was less than CD4^+^ cells, but interestingly a significant amount of IFN-γ was produced by CD8^+^ T cells in cell culture and around 5–12% of CD8^+^ cells was positive for IFN-γ secretion by ICS assay. It is concluded that CD8^+^ T cells contribute along with CD4^+^ Th1 cells in IFN-γ production in individuals with history of CL. Despite the limited reports of CD4^+^ Th1 cells as the main source of IFN-γ production in CL patients [47], [48] in most studies of CD4^+^ Th1/Th2 paradigm in human CL, PBMCs rather than purified T cells were used, hence the role of IFN-γ producing CD8^+^ T cells should not be ruled out when reporting a “Th1” type response in PBMC culture.

The strong lymphoproliferative and IFN-γ response in self healing CL caused by *L. braziliensis* is previously shown [49], [50]. In the current study, HCL volunteers with spontaneous healing during 1.5–5 months were recruited. Individuals with history of self healing CL are presumed to be protected against further *Leishmania* infection. The blood samples were collected a few months to years after cure of CL lesions. The strong LST response (mean LST = 10.7±7.5 mm) and IFN-γ production is an indication of sustaining cell mediated immune response. This sustaining protective immunity is mediated not only through the expansion of antigen-specific IFN-γ producing CD4^+^ Th1 cells, but also through IFN-γ producing CD8^+^ T cells. The question that which one of these T cell subsets plays a more important role in IFN-γ production at the initiation of exposure to sand fly bite needs to be explored.
